# Digital and social media use among adolescents in Arctic Norway: Exploring risk and protective factors in Finnmark County

**DOI:** 10.1016/j.dialog.2025.100212

**Published:** 2025-04-01

**Authors:** Shiho Hansen

**Affiliations:** Sami Norwegian National Advisory Unit for Mental Health and Substance Use, Finnmark Hospital Trust, Norway

**Keywords:** Arctic, Norway, Adolescent health, Digital, Social media, Risk factor, Protective factor, PLS-SEM

## Abstract

Digital and social media use among adolescents in Finnmark County, Arctic Norway, has increased significantly over the past decade. However, the mechanisms linking risk and protective factors to these behaviors remain underexplored. This study examines the associations between individual, environmental, and behavioral factors influencing digital and social media use among high school students in Finnmark. Using data from the 2021 Ungdata survey, which included responses from 2086 high school students in Finnmark County, the research examined associations between individual factors, environmental influences (e.g., family, peers, schools), and risk behaviors (e.g., substance use, antisocial behavior) through partial least squares structural equation modeling with stepwise regression. The findings revealed distinct patterns: digital media use was associated with environmental factors such as relationships with parents, school connectedness, and satisfaction with the local area. In contrast, social media use was associated with peer dynamics, internal feelings, and negative experiences, including depression and sexual harassment. While media use fostered social connections, heavy use was associated with reduced physical activity and face-to-face interactions, exacerbating regional challenges like geographic isolation and cultural diversity. These findings underscore the importance of distinguishing between digital and social media in research and interventions, integrating the unique sociocultural context of Finnmark. Future studies should explore the behavioral dimensions of media use and incorporate ethnicity-related factors to deepen the understanding of adolescent media behaviors in Arctic regions.

## Highlights

• Digital media links to family, school ties, and local area satisfaction.
• Social media ties to peer dynamics, bullying, depression, and harassment.
• Regional factors in Finnmark may shape adolescents' media use behaviors.
• Future studies should explore media intensity and sociocultural factors in Finnmark.

## 1 Background

Over the past decade, the use of social media and digital platforms has become central to the lives of adolescents and young adults [1]. Digital media use influences various public health outcomes, including mental health, health behaviors, and access to health information, and it can bring both benefits and risks to adolescents that have substantial public health implications [2,3]. The benefits include early learning, exposure to new ideas and knowledge [4], and helping teens connect with peers they know in real life [5] and build relationships with individuals from diverse backgrounds across the world [6]. However, the risks—such as mental health challenges, disrupted sleep, exposure to harmful content, substance use, and cyberbullying—pose significant public health concerns [3,4,7]. For instance, excessive reliance on technology for socializing can reduce face-to-face interactions, limiting meaningful connections with family and friends [2]. Furthermore, exposure to violent media, such as video games, can desensitize teens to violence and increase aggression, while prosocial media can foster empathy and altruism [8]. A systematic review found that adolescents who spend more time on social media are at greater risk for mental health issues, partly due to cyberbullying and unrealistic social comparisons [9].

Cyberbullying, in particular, is a pressing public health challenge. Adolescents' constant engagement with digital platforms heightens the risk of cyberbullying, leading to adverse consequences such as anger, low self-esteem, and even suicidal thoughts [10]. The Health Behavior in School-aged Children (HBSC) study, which surveyed nearly 280,000 young people aged 11, 13, and 15 across 44 countries and regions in Europe, Central Asia, and Canada in 2022, revealed a sharp rise in problematic social media use, including cyberbullying, among adolescents [11]. Given these concerns, integrating a public health perspective is essential when examining adolescents' digital behaviors. Understanding the interplay between individual factors, environmental influences (family, school, peers), and regional contexts—as seen in Finnmark, Norway—can provide insights into how digital and social media shape adolescent health outcomes.

## 2 Digital and social media use and environmental influences

Due to the relatively new nature of this research area, studies examining the interplay between adolescents' digital and social media use and environmental influences, such as parents, peers, and schools, are steadily increasing. Family dynamics, including parent-child relationships and media habits, significantly influence children's media use behaviors [12,13]. For example, research on parental influence shows that closer parent-child relationships and open communication reduce PIU (Problematic Internet Use) symptoms, whereas protective parenting yields mixed results [14]. Both parents contribute individually to their adolescent child's safe Internet use [15]. Additionally, heavy social media use is associated with negative parent–child relationships [16]. Findings also reveal that parents “friending” their children on Facebook reduces conflict and strengthens closeness, particularly in strained relationships, without raising privacy concerns [17].

Peer influence also plays a pivotal role in shaping adolescents' social media behaviors. For instance, peer communication pressure significantly impacts adolescents' online social behaviors [13]. Peer influence contributes to the daily rhythms of social media use, driving clustering effects among individuals with similar interaction behaviors [18]. Peer interactions are also central to the development and spread of Internet and gaming addictions [18]. Friendship quality and social support predict IU (Internet Use) and PIU differently—higher-quality friendships enhance IU, while poor support correlates with PIU [19,20].

Both parents and peers play a role in reducing risky social media behaviors, but their impact on problematic use is minimal, with low self-control emerging as the primary driver [21]. While peers often promote risky online behavior and internet addiction, active parental involvement can counterbalance these effects, demonstrating that engaged parents exert greater influence than peers [22].

The classroom environment is another significant factor in moderating PIU among adolescents [14]. Shared gaming interests in classrooms have been shown to promote social connections, reduce symptoms of PIU, and decrease feelings of isolation [23]. Moreover, perceived teacher autonomy and opportunities for decision-making lower tendencies for online gaming. These effects are mediated by psychological satisfaction and school engagement, highlighting the role of supportive educational contexts in fostering healthy Internet use [24].

## 3 Digital and social media use in Norway and Finnmark County

In Norway, 79 % of male adolescents and 89 % of female adolescents aged 11–17 did not meet the World Health Organization's recommended levels of physical activity in 2022 [25]. Additionally, 97 % of young people aged 16–24 had used social media in the last three months [26]. Adolescents in Finnmark County, located in the Arctic region at the northern tip of Norway, have increasingly spent time on digital devices, such as social media and online games, while the time dedicated to physical activities, such as walking and jogging, has declined over the last decade [27]. Furthermore, compared to the national average, high school students in Finnmark spend more time on digital devices at home and allocate less time to physical exercise [27].

Several studies in Norway have examined the impact of media use. For instance, a study in the Oslo metro area found that digital media use was associated with increased aggressive behaviors (AGGs) [28]. However, in Finnmark, digital and social media use among high school students was primarily linked to increased alcohol consumption and smoking, but not to AGGs [29]. Despite these findings, there is limited understanding of the interplay between adolescents' digital and social media use and environmental influences, such as parents and peers, in Finnmark.

Finnmark is the northernmost county in Norway, located in the Arctic region. Compared with other regions of the country, Finnmark has a small, dispersed, and isolated population. The county also has a distinct sociocultural and context, including the Sámi (Sámi-speaking peoples inhabiting the Sápmi region) and the Kven (descendants of people who migrated to Northern Norway from Northern Finland and Northern Sweden multiethnic in the 18th and 19th centuries), as well as people from Northern Finland and Northern Sweden, and intermarriage among these groups [30].

Several studies on digital media use have emphasized the role of cultural differences (for example, individualism in Western cultures and collectivism in Asian cultures) in shaping privacy behaviors, social media engagement, and adoption patterns across regions [[31], [32], [33]]. However, most of this research has not been conducted in Norway or the Arctic region.

The distinctive geographical and sociocultural context of Finnmark provides a critical backdrop for analyzing adolescents' media use, with several key factors warranting consideration. First, demographic and lifestyle factors can play a significant role for adolescents in Finnmark exhibit higher engagement with digital devices and lower levels of physical activity compared to the national average. These behaviors contribute to region-specific trends, such as reduced face-to-face interactions. Second, **cultural diversity** shapes adolescent behaviors in unique ways. The multiethnic composition of Finnmark, with its blend of Sámi, Kven, and other Nordic influences, shapes social and cultural norms around technology use. This diversity offers a unique lens through which to study adolescent behaviors. Finally, **the interplay of environmental influences**, such as family and peer dynamics, highlights the impact of cultural and social norms in the region. These factors may mediate adolescent behaviors differently compared to urban centers in Norway or other countries. Additionally, classroom environments and school engagement within Finnmark communities play a pivotal role in shaping patterns of problematic internet use (PIU), with these effects likely moderated by the region's unique cultural and contextual factors.

## 4 Summary of previous research and study gap

The increasing use of digital and social media among adolescents has significant public health implications, influencing mental health, social behaviors, and risk behaviors. Existing research highlights both benefits (e.g., social connectivity, knowledge acquisition) and risks (e.g., cyberbullying, substance use, and reduced face-to-face interactions). While studies have explored the role of individual and environmental factors—such as family, peers, and schools—in shaping media use patterns, there remains limited research on how these factors interact within unique sociocultural and geographic contexts such as Finnmark County. Most existing studies predominantly focus on urban settings or culturally homogeneous regions. The unique characteristics of Finnmark—its Arctic geography, sparse population, and diverse sociocultural fabric—necessitate targeted research to understand how family, peers, and schools interact to influence adolescents' digital behaviors.

### 4.1 Theoretical and contextual frameworks

This study is grounded in Bronfenbrenner's Ecological Systems Theory [34], which highlights the interconnectedness of individual, relational, and environmental factors in human development. This framework is particularly relevant to understanding adolescents' digital media use, as it allows for an examination of the interplay between personal behaviors, family dynamics, peer interactions, and broader institutional influences such as schools and communities. In Finnmark, the interplay of family, peers, school, and community is examined within this framework. The region's sociocultural uniqueness may shape distinct patterns of risk and protective factors influencing adolescents' media use compared to other parts of Norway or internationally.

### 4.2 Purpose of the study

This study aims to investigate the underlying mechanisms linking risk and protective factors with digital and social media use among high school students in Finnmark County. Specifically, it explores how individual characteristics (e.g., socioeconomic status, depressive thoughts, negative experiences, physical activity), environmental factors (e.g., family, school, peers, local area), and risk behaviors (e.g., substance use, rule-breaking, aggression, bullying) are associated with digital and social media use.

## 5 Methods

### 5.1 Data source and samples

This study used cross-sectional data from the Ungdata survey. Ungdata is a national data collection scheme on health, well-being, and risk behaviors among adolescents in Norway [35]. Norwegian Social Research (NOVA) is responsible for overall coordinating, while the Regional Drug and Alcohol Competence Centers (KoRus) are responsible for conducting the surveys at the municipal level. Since 2014, the survey has been conducted every three years in both junior high and high schools across Norway. The questionnaire consists of a core module, which remains consistent across all survey waves, along with optional questions that municipalities can select based on their specific interests and needs. The questionnaires cover various aspects of adolescents' lives, such as free time use, physical activity, social media use, and environmental factors surrounding adolescents, including family, friends, school, and neighbors. Additional areas included substance use, anti-social behaviors, depressive thoughts, and perceptions of health and future [36].

The surveys were administered anonymously online during school hours. For further details on Ungdata, refer to their website [35]. This study used the data collected in 2021 from high school students in Finnmark. The response rate was 73 % [37]. Reasons for non-response included student absence on the day of the survey or a refusal to participate. A total of 2129 high school students in Finnmark joined the survey. Observations from participants who answered only the first few questions were excluded from the analysis, resulting in a final sample of 2086 high school students.

The distribution of participants by grade level was as follows: 919 (44.1 %) in VG1 (1st year of high school), 737 (35.3 %) in VG2 (2nd year), 422 (20.2 %) in VG3 (3rd year), and 8 (0.4 %) with missing grade information. Among them, 1066 (51.1 %) were female, 978 (46.9 %) were male, and 42 (2.0 %) had missing gender data. The dataset was obtained from the Survey Bank of the Norwegian Agency for Shared Services in Education and Research, or Sikt [38].

### 5.2 Partial least squares structural equation modeling

This study utilized Partial Least Squares Structural Equation Modeling (PLS-SEM) to examine the relationships between exogenous and endogenous variables. PLS-SEM is a robust multivariate statistical technique commonly applied in disciplines such as the social sciences and health research. Structural Equation Modeling (SEM) encompasses two primary analytical approaches: covariance-based SEM (CB-SEM) and PLS-SEM.

A key advantage of PLS-SEM lies in its flexibility regarding data characteristics and model complexity [39]. Unlike CB-SEM, PLS-SEM is particularly well-suited for studies with small to medium sample sizes. Additionally, it does not require the data to conform to a normal distribution, enabling effective analysis of non-normal, skewed, or kurtotic datasets. PLS-SEM is also adept at estimating complex models comprising multiple constructs, indicators, and interrelationships, without imposing strict statistical assumptions.

Another notable strength of PLS-SEM is its robustness to measurement issues [39]. It demonstrates greater tolerance for measurement errors in observed indicators compared to CB-SEM, thereby enhancing the reliability of results even when data quality is less than optimal. Furthermore, PLS-SEM employs bootstrapping techniques to evaluate the significance of path coefficients within the model. This non-parametric method facilitates rigorous statistical testing without relying on distributional assumptions, providing researchers with greater confidence in their findings.

### 5.3 Sample size

A traditional rule-of-thumb method for estimating the sample size in PLS-SEM is the “10-times rule,” which suggests that the sample should be at least 10 times the number of estimated parameters. However, this approach may lead to inaccuracies in complex models. More precise methods, such as the inverse square root method and the gamma-exponential method, have been proposed [40]. The inverse square root method is widely used due to its simplicity. Assuming a common power level of 80 % and a significance level of 5 %, the minimum sample size is calculated as follows: N > (2.486/(|β|_min_))^2^ In social science studies, it is often acceptable to have low path coefficients (for example |β|_min_ is 0.1) that are still statistically significant when using bootstrapping techniques [41]. Assuming a minimum path coefficient |β|_min_ 0.1, the required sample size is: and N > (2.486/0.1))^2^ = 618.02. Thus, the minimum sample size required for analysis will be 619, assuming |β|_min_ is more than 0.1.

### 5.4 Measures

This study utilized questions from both the core module and optional questions. Questions that were not asked of all grades were excluded from the analysis. In total, 74 variables were used for data analysis.

A reverse coding system was employed in this study, wherein indicators were assigned higher values if they represented undesirable content or phenomena. For example, a variable related to school, “I often don't want to go to school [Jeg gruer meg ofte til å gå på skolen],” was categorized into four levels: 4 = totally agree [helt enig] and 1 = totally disagree [helt uenig]. In this case, a value of 1 was considered undesirable.

### 5.5 Model development

To conduct the PLS-SEM analysis, a hypothetical model was developed that integrates both measured and latent variables. In PLS-SEM, latent variables represent unobserved constructs derived from multiple measured variables, allowing for a nuanced understanding of complex relationships.

This model is grounded in Bronfenbrenner's Ecological Systems Theory [34], which emphasizes the dynamic interplay between individual factors, environmental influences, and risk behaviors. This theoretical framework offers a holistic perspective on how multiple layers of an adolescent's environment interact to shape behaviors. To further strengthen the rationale for variable selection, relevant empirical studies [[12], [13], [14], [15], [16], [17], [18],[21], [22], [23], [24],^29^] and the Ungdata strategy [36] were incorporated. The developed model comprises 27 latent variables. A complete list of latent variables and their corresponding measured variables is provided in Table 1.

**Table 1:** Latent variables and their corresponding measured variables (questionnaire items).

latent variables		measured variable	questionnaire items
socioeconomic status	SES	Ses	socioeconomic status
Religion	RELIG	Relig	How much does religion mean for how you live your daily life?
Positivity	POSITIV	positiv1	my life is good.
		positiv2	I like myself the way I am.
		positiv3	On a scale from 0 to 10, how satisfied are you with your life these days?
quality of life	KVAL	kval1	been happy.
		kval2	been engaged
		kval3	been optimistic about the future
		kval4	felt that you master things
future outlook	FRAM	fram1	Do you think that you will study at a university or university college?
		fram2	Do you think that you will have a good and happy life?
		fram3	Do you think that you will complete upper secondary school?
depressive thoughts	DEPR	During the past week, have you been affected by any of the following issues	
		depr1	felt that everything is a struggle
		depr2	felt unhappy, sad or depressed
		depr3	felt hopelessness about the future
		depr4	felt stiff or tense
		depr5	worried to much about things
feeling of performance pressure	PRESS	press1	pressure to look good or have a nice body.
		press2	pressure to do well at school.
		press3	pressure to do well in sports
		press4	pressure to have many followers and likes on social media.
		press5	Have you experienced so much pressure in the past week that you have had trouble coping with it?
physical activity	FYSISK	fysak1	How often do you do physical activity whick gets you out of breath or makes you sweaty?
		fysak2	How often do you participate in other kinds of organised physical activity (dance, martial arts, etc.)?
		fysak3	Think about the last seven days. How many days were you physically active to the point of getting out of breath or sweating for at least 60 min in a day?
Sport	SPORT	Sport	ride a skateboard, snowboard, longboard, street dance, climb, do parkour, or similar activities (not in a sports club).
COVID-19	COVID	covid1	Has the coronavirus pandemic negatively affected your life?
		covid2	I have worried more than before the corona pandemic
		covid3	I have felt more unhappy, sad, or depressed than before the coronavirus.
		covid4	I have participated in fewer leisure activities than before the corona pandemic
		covid5	I have felt more lonely than before the corona pandemic
		covid6	There has been more arguing in my family than before the coronavirus.
being bullied	MOBB	mobb1	Are you sometimes teased, threatened or frozen out by other young people at school or in your free time?
		mobb2	Are you bullied, threatened, or excluded online?
		mobb3	Has another young person threatened, attacked, or robbed you with objects or weapons?
sexual harassment	SEXTRAK	sextrak1	Someone touched you in a sexual way against your will.
		sextrak2	Someone hurtfully called you a whore, gay, or other words with sexual content.
		sextrak3	Someone spread negative sexual rumors about you.
relationships with parents	FAM1	fam1	My parents usually know where I am and who I am with in my free time.
		fam2	My parents know most of the friends I hang out with in my free time.
		fam3	My parents are very interested in my life.
poor relationships with parents	FAM2	Rfam	I try to keep most of my free time activities hidden from my parents.
Peers	VENN	venn1	Do you have at least one friend who you trust completely and who you can tell absolutely anything?
		venn2	Do you have someone to be with during your free time?
		venn3	Do you have someone to be with during recess at school?
being with peers	BVENN1	Bvenn	I spent most of the evening out with friends.
school	SKOLE	skole1	My teachers care about me
		skole2	I get stressed by schoolwork
local area	NAER	Think about the areas around where you live. How good do you think that services for young people are in terms of:	
		naer1	sports facilities
		naer2	culture (cinemas, concert veneus, libraries, etc.)
local safety	NAERT	Naert	When you are out in the evening, do you feel safe in the local area where you live?
drinking	BALKO	balko1	Do you ever drink any kinds of alcoholic drinks?
		balko2	How old were you the first time you drank so much alcohol?
		balko3	Had so much to drink that you felt clearly intoxicated
smoking	BROEYK	broeyk1	Do you smoke?
		broeyk2	Do you use snus (tobacco that you put under your lip)?
		broeyk3	Do you use e-cigarettes/vape?
drug use	BDRUG	bdrug1	used hash/maijuana/cannabis
		bdrug2	used other drugs
		bdrug3	Over the past year (the past 12 months), have you been offered cannabis (hash or marijuana)?
rule-breaking behavior	BANTI	banti1	not paid for the cinema, sporting events, bus and train tickets, etc. when you sholud have paid
		banti2	spent the whole night away from home without your parents knowing where you were
		banti3	skipped school
		banti4	consciously cheated on a test or assignment that was graded
aggressive antisocial behavior	BANTIV	bantiv1	deliberately damaged or broken window panes, bus seats, post boxes, etc. (carried out vandalism)
		bantiv2	illegally spray-painted or tagged walls, buildings, trains, buses, etc.
		bantiv3	carried a knife or other weapons in places where it is not allowed.
		bantiv4	hacked, scammed someone, or engaged in other online criminal activities.
		bantiv5	sold hash or other illegal substances.
bullying others	BMOBB	bmobb	Do you sometimes take part in teasing, threatening og freezing out other young people at school or in your free time?
digital use	DIGI	How much time to you spend on the following things:	
		digi1	playing computer games/video games
		digi2	watch movies/series/YouTube
social media use	SMS	sms1	spent most of the evening socializing on the Internet or by mobile phone (talking, chatting, etc.)
		sms2	How much time to you spend on social media (Facebook, Instagram, etc.)?

### 5.6 Structure of the model

The developed model for the PLS-SEM analysis is structured around the distinction between endogenous and exogenous latent variables, aligning with standard practices in structural equation modeling [39]. PLS-SEM uses the term “endogenous variables” instead of dependent variable and the term “exogenous variable” for independent variable [39]. This structure allows for a comprehensive examination of how various personal, social, and environmental factors influence adolescents' digital and social media use.

The principal endogenous variables for the PLS-SEM analysis were digital media use (DIGI) and social media use (SMS). As shown in Table 1, DIGI refers to the amount of time spent playing on a computer/video games and watching movies, series, or YouTube videos, as measured by the variables “digi1” and “digi2.” SMS encompasses the amount of time spent on social interactions via platforms such as Facebook, Instagram, and whether the participant spends most of the evening socializing on the Internet or via mobile phone (talking, chatting, etc.), as measured by “sms1” and “sms2” (see Table 1). Tables 2a, 2b, 2c present the descriptive statistics for the measured variables related to the endogenous constructs.

**Table 2a:** Descriptive Statistics (digi1 & digi2).

	Frequency (Percent)	
	digi1 (playing computer games/video games)	digi2 (watch movies/series/YouTube)
No time	863 (41.4 %)	74 (3.5 %)
Less than 30 min.	160 (7.7 %)	151 (7.2 %)
30 min – 1 h	158 (7.6 %)	351 (16.8 %)
1–2 h	231 (11.1 %)	499 (23.9 %)
2–3 h	172 (8.2 %)	396 (19 %)
More than 3 h	334 (16 %)	463 (22.2 %)
No answer	168 (8.1 %)	152 (7.3 %)

**Table 2b:** Descriptive statistics (sms1).

sms1 (spent most of the evening socializing on the Internet)	Frequency (Percent)
Never	179 (8.6 %)
Once	306 (14.7 %)
2–5 times	643 (30.8 %)
6 times or more	837 (40.1 %)
No answer	121 (5.8 %)

**Table 2c:** Descriptive statistics (sms2).

sms2 (spend on social media (Facebook, Instagram, etc.))	Frequency (Percent)
No time	37 (1.8 %)
Less than 30 min.	126 (6 %)
30 min – 1 h	181 (8.7 %)
1–2 h	319 (15.3 %)
2–3 h	405 (19.4 %)
More than 3 h	869 (41.7 %)
No answer	149 (7.1 %)

Other latent variables were treated as exogenous variables. Socioeconomic status (SES) serves as a foundational contextual factor, influencing access to digital devices and opportunities for extracurricular activities. Individual factors, including religion (RELIG), positivity (POSITIV), quality of life (KVAL), future outlook (FRAM), depressive thoughts (DEPR), and feelings of performance pressure (PRESS), capture emotional and psychological aspects that may affect media use patterns. For example, depressive thoughts may lead to increased screen time as a coping mechanism, while a positive outlook may moderate excessive use.

The model also considers how adolescents allocate their free time, with variables like physical activity (FYSISK) and sports participation (SPORT) reflecting engagement in offline activities that may counterbalance screen time. Negative experiences, such as being bullied (MOBB) or sexually harassed (SEXTRAK), are included due to their potential to increase online engagement as a form of social compensation or escape. COVID-19-related experiences (COVID) are incorporated to capture the unique impact of the pandemic on adolescents' daily lives, particularly the increased reliance on digital platforms for education and social interaction.

Environmental factors, including parental influence (FAM1, FAM2), peer relationships (VENN, BVENN), school environment (SKOLE), and perceptions of the local community (NEAR, NEART), provide insight into the social contexts that shape media use. Risk behaviors such as substance use—alcohol (BALKO), smoking (BROEYK), and drug use (BDRUG)—alongside antisocial behaviors, including non-aggressive rule-breaking (BANTI), aggressive behavior (BANTIV), and bullying others (BMOBB), are included to examine potential co-occurring behaviors that may influence digital media consumption patterns.

During factor analysis, some latent variables—such as those related to parental influence, physical activities, and digital use—were found to exhibit two distinct dimensions, which were incorporated into the model to capture the complexities of these constructs.

### 5.7 Data analysis

PLS-SEM analysis was performed to investigate the associations between exogenous and endogenous variables. As the first step of the PLS-SEM analysis, model evaluation was conducted for both the measurement and structural models. Assessing the measurement models included evaluating factor loadings, reliability, convergent validity, discriminant validity, and indicator collinearity.

Factor loadings indicate the variance explained by a measured variable on a specific factor, with factor loadings of 0.5 or higher considered acceptable. Internal consistency reliability was evaluated using composite reliability (CR) for each latent variable. While Cronbach's alpha is frequently used for assessing internal consistency, it is known to be conservative and assumes equal indicator loadings across the population. Composite reliability, on the other hand, offers more flexibility regarding loading assumptions and is thus more suitable for PLS-SEM analysis [39], CR values of 0.7 or higher were considered acceptable.

The average variance extracted (AVE) was employed to evaluate the convergent validity of the latent variables, with a threshold of 0.4 or higher indicating adequate convergence. Convergent validity ensures that indicators of a specific construct are highly correlated, confirming that they accurately represent the same underlying concept. In contrast, discriminant validity was assessed using the heterotrait–monotrait (HTMT) ratio of correlations, which examines the extent to which a reflectively measured construct is distinct from other constructs within the model. An HTMT value exceeding 0.90 indicates a lack of discriminant validity, suggesting that the constructs may not be sufficiently distinct from one another [42]. For the structural models, collinearity among the predictor constructs was examined using the variance inflation factor (VIF). Although VIF values of 5.0 or higher suggest collinearity issues, a more conservative threshold of 3.3 was adopted [43].

Subsequently, PLS-SEM regression analysis was conducted to examine the relationships between exogenous and endogenous variables. To ensure the robustness of parameter estimates, bootstrapping with 10,000 resamples was employed to generate confidence intervals [39]. A stepwise regression method was employed, wherein the initial model included all potential explanatory variables, and non-statistically significant factors were progressively removed.

Prior to conducting stepwise regression, the sample was divided into two groups based on gender (boys and girls) to allow separate regression analyses for each group. The path coefficients for each group were then compared using multi-group analysis in PLS-SEM (PLS-MGA). Results indicated that 98.1 % (51 out of 52 paths in total) of the path coefficients showed no statistically significant differences between the two groups. Results indicated that 98.1 % (51 out of 52 paths in total) of the path coefficients showed no statistically significant differences between the two groups. The same procedure was applied to three school grades (10th–12th grades) using PLS-MGA. Results showed no statistically significant differences between the different grades, with 94.1 % (10th vs. 11th), 96.1 % (10th vs. 12th), and 96.1 % (11th vs. 12th) of the path coefficients showing no significant variation (48, 49, and 49 out of 51 paths, respectively). This result suggests that PLS-SEM analysis can be applied across genders [39].

The present study used SPSS Statistics 27 (IBM Corp., Armonk, N.Y., USA) to perform descriptive statistics, while R 4.4.1 (The R Foundation for Statistical Computing, Vienna, Austria) with the SEMinR package (version 2.3.3) was used for PLS-SEM analysis. The significance threshold was set at *p* < 0.05.

### 5.8 Ethics

This study used the anonymized databases from Ungdata survey that has been approved by Norwegian Centre for Research Data (NSD). The survey was conducted in accordance with the Declaration of Helsinki. Participation was voluntary and informed consent was obtained from all students prior to the survey. Ethical issues were thus addressed by the institutions conducting the surveys and a separate ethics approval for this study was not necessary.

## 6 Results

### 6.1 Evaluation of measurement models

Table 3 presents all variables used in this study and the values for assessing the measurement model. While some factor loadings were below 0.50, they were deemed acceptable since their values were statistically significant [39]. The composite reliability (CR) and average variance extracted (AVE) values were within acceptable ranges, with CR being 0.70 or higher and AVE being 0.40 or higher. For the structural model assessment (Table 4), all variance inflation factor (VIF) values were within an acceptable range (less than 3.30).

**Table 3:** Values for assessing the measurement model.

latent variables	measured variables	Mean	Std.Dev.	loadings	rhoC	AVE
SES	Ses	1.844	0.510	1.000	1.000	1.000
RELIG	Relig	3.497	0.806	1.000	1.000	1.000
POSITIV	positiv1	1.565	0.767	0.715	0.828	0.625
	positiv2	1.927	0.951	0.861		
	positiv3	4.018	1.953	0.569		
KVAL	kval1	2.330	0.820	0.397	0.786	0.498
	kval2	2.654	0.916	0.581		
	kval3	2.645	1.139	0.616		
	kval4	2.662	1.028	0.878		
FRAM	fram1	1.746	0.69	0.889	0.682	0.444
	fram2	1.370	0.543	0.603		
	fram3	1.075	0.295	0.376		
FYSISK	fysisk1	2.631	1.286	0.667	0.772	0.542
	fysisk2	3.990	1.641	0.9		
	fysisk3	3.568	1.093	0.543		
SPORT	Sport	5.526	0.934	1.000	1.000	1.000
DEPR	depr1	2.398	1.019	0.803	0.817	0.486
	depr2	2.166	1.008	0.510		
	depr3	2.074	1.034	0.455		
	depr4	2.168	0.987	0.717		
	depr5	2.517	1.057	0.850		
PRESS	press1	2.560	1.383	0.887	0.816	0.485
	press2	3.097	1.324	0.654		
	press3	2.101	1.287	0.483		
	press4	1.614	1.035	0.834		
	press5	1.736	0.856	0.491		
FYSISK	fysisk1	2.631	1.286	0.667	0.772	0.542
	fysisk2	3.990	1.641	0.9		
	fysisk3	3.568	1.093	0.543		
SPORT	Sport	5.526	0.934	1.000	1.000	1.000
COVID	covid1	2.581	1.214	0.674	0.810	0.424
	covid2	1.951	0.957	0.822		
	covid3	1.915	1.001	0.682		
	covid4	2.249	1.162	0.502		
	covid5	1.905	1.026	0.549		
	covid6	1.381	0.756	0.475		
MOBB	mobb1	1.518	1.077	0.358	0.707	0.468
	mobb2	1.332	0.873	0.747		
	mobb3	1.053	0.325	0.773		
SEXTRAK	sextrak1	1.290	0.677	0.708	0.805	0.585
	sextrak2	1.396	0.861	0.617		
	sextrak3	1.347	0.745	0.907		
FAM1	fam1	1.625	0.773	0.908	0.848	0.655
	fam2	1.680	0.812	0.796		
	fam3	1.633	0.721	0.634		
FAM2	rfam	1.991	0.919	1.000	1.000	1.000
VENN	venn1	1.569	0.786	0.776	0.810	0.591
	venn2	1.674	0.698	0.816		
	venn3	1.443	0.652	0.610		
BVENN1	bvenn	2.183	1.017	1.000	1.000	1.000
SKOLE	skole1	1.866	0.766	0.37	0.679	0.561
	skole2	3.570	1.199	0.988		
NAER	naer1	2.392	1.105	0.928	0.782	0.659
	naer2	2.665	1.223	0.527		
NAERT	Naert	1.508	0.715	1.000	1.000	1.000
BALKO	balko1	2.768	1.239	0.786	0.816	0.562
	balko2	3.360	2.053	0.799		
	balko3	2.555	1.520	0.938		
BROEYK	broeyk1	1.631	1.050	0.692	0.815	0.603
	broeyk2	1.803	1.433	0.598		
	broeyk3	1.319	0.721	0.92		
BDRUG	bdrug1	1.159	0.647	0.732	0.834	0.630
	bdrug2	1.086	0.507	0.585		
	bdrug3	1.326	0.659	0.751		
BANTI	banti1	1.579	1.123	0.593	0.777	0.468
	banti2	1.701	1.192	0.612		
	banti3	1.876	1.202	0.619		
	banti4	1.591	0.939	0.751		
BANTIV	bantiv1	1.118	0.504	0.548	0.776	0.423
	bantiv2	1.062	0.401	0.503		
	bantiv3	1.109	0.547	0.739		
	bantiv4	1.057	0.396	0.745		
	bantiv5	1.049	0.399	0.384		
BMOBB	bmobb	1.295	0.830	1.000	1.000	1.000
DIGI	digi1	2.839	1.976	0.994	0.672	0.559
	digi2	4.231	1.396	0.352		
SMS	sms1	3.088	0.969	0.344	0.674	0.560
	sms2	4.826	1.360	0.996		

Notes: rhoC: Composite reliability used to assess internal consistency reliability. Values of 0.7 or higher were considered acceptable.

AVE: Average variance extracted, used to assess the validity of the latent variables, with a threshold value of 0.4 or higher.

**Table 4:** Discriminant validity: Heterotrait-monotrait Ratio Statistics (HTMT).

	SOE	RELIG	POSITIV	KVAL	FRAM	DEPR	PRESS	FYSISK	SPORT	COVID	MOBB	SEXTRAK	FAM1
RELIG	0.038												
POSITIV	0.143	0.076											
KVAL	0.108	0.096	0.845										
FRAM	0.184	0.092	0.635	0.621									
DEPR	0.073	0.040	0.735	0.719	0.575								
PRESS	0.033	0.050	0.520	0.429	0.319	0.666							
FYSISK	0.252	0.072	0.176	0.246	0.423	0.154	0.178						
SPORT	0.046	0.037	0.065	0.117	0.078	0.065	0.050	0.158					
COVID	0.057	0.052	0.346	0.303	0.254	0.451	0.520	0.112	0.033				
MOBB	0.049	0.082	0.263	0.176	0.398	0.224	0.270	0.059	0.097	0.170			
SEXTRA	0.018	0.016	0.303	0.223	0.284	0.377	0.393	0.042	0.018	0.273	0.623		
FAM1	0.192	0.087	0.394	0.341	0.365	0.201	0.097	0.189	0.047	0.154	0.200	0.226	
FAM2	0.087	0.006	0.170	0.127	0.272	0.121	0.074	0.152	0.015	0.096	0.177	0.173	0.333
VENN	0.134	0.042	0.517	0.461	0.494	0.359	0.198	0.248	0.042	0.186	0.408	0.150	0.330
BVENN1	0.000	0.034	0.101	0.127	0.176	0.029	0.064	0.135	0.059	0.075	0.040	0.165	0.061
SKOLE	0.031	0.032	0.628	0.708	0.444	0.743	0.785	0.078	0.112	0.505	0.309	0.318	0.252
NAER	0.151	0.023	0.163	0.166	0.225	0.179	0.131	0.135	0.004	0.125	0.187	0.142	0.186
NAERT	0.071	0.076	0.236	0.209	0.265	0.242	0.217	0.125	0.039	0.157	0.244	0.191	0.123
BALKO	0.062	0.126	0.132	0.058	0.135	0.220	0.227	0.089	0.016	0.237	0.253	0.479	0.210
BROEYK	0.152	0.031	0.139	0.047	0.234	0.149	0.073	0.183	0.055	0.134	0.407	0.368	0.294
BDRUG	0.090	0.069	0.118	0.050	0.330	0.129	0.101	0.048	0.118	0.155	0.459	0.301	0.254
BANTI	0.081	0.012	0.264	0.219	0.373	0.301	0.275	0.114	0.082	0.192	0.405	0.565	0.397
BANTIV	0.039	0.084	0.118	0.072	0.398	0.079	0.084	0.042	0.044	0.074	0.627	0.300	0.205
BMOBB	0.035	0.057	0.013	0.045	0.116	0.039	0.062	0.076	0.067	0.046	0.578	0.257	0.066
SMS	0.076	0.059	0.123	0.113	0.104	0.294	0.300	0.027	0.065	0.225	0.088	0.258	0.029
DIGI	0.155	0.139	0.139	0.157	0.434	0.211	0.271	0.334	0.127	0.169	0.118	0.141	0.264


	FAM2	VENN	BVENN1	SKOLE	NAER	NAERT	BALKO	BROEYK	BDRUG	BANTI	BANTIV	BMOBB	SMS
VENN	0.144												
BVENN	0.005	0.393											
SKOLE	0.070	0.284	0.040										
NAER	0.085	0.216	0.013	0.325									
NAERT	0.067	0.219	0.045	0.247	0.118								
BALKO	0.094	0.177	0.302	0.222	0.108	0.063							
BROEYK	0.182	0.070	0.227	0.151	0.115	0.064	0.636						
BDRUG	0.152	0.061	0.086	0.080	0.051	0.086	0.464	0.573					
BANTI	0.268	0.062	0.247	0.360	0.107	0.095	0.651	0.574	0.489				
BANTIV	0.146	0.133	0.065	0.147	0.069	0.143	0.275	0.426	0.614	0.576			
BMOBB	0.135	0.048	0.068	0.095	0.054	0.076	0.187	0.259	0.295	0.335	0.358		
SMS	0.042	0.177	0.229	0.277	0.076	0.012	0.260	0.104	0.062	0.223	0.083	0.046	
DIGI	0.241	0.204	0.180	0.273	0.157	0.067	0.103	0.220	0.164	0.099	0.125	0.070	0.432

Note: An HTMT value exceeding 0.90 indicates a lack of discriminant validity, suggesting that the constructs may not be sufficiently distinct from one another.

### 6.2 Evaluation of structural models

#### 6.2.1 Digital media use

Table 5 presents the path coefficients and *p*-values for the structural model. In the final model incorporating individual factors, digital media use was significantly positively associated with religion (RELIG, *β* = 0.068), future outlook (FRAM, *β* = 0.176), and sport activity (SPORT, *β* = 0.106). Conversely, it was negatively associated with pressure (PRESS, *β* = −0.157), physical activity (FYSISK, *β* = −0.095), and sexual harassment (SEXTRAK, *β* = −0.094). No significant associations were found with depressive thoughts (DEPR), COVID-19 (COVID), or being bullied (MOBB).

**Table 5:** Path coefficients and *p*-values for the structural model.

	Digital media (DIGI)						Social media (SMS)					
	Initial model			Final model			Initial model			Final model		
	VIF	*β*	P	VIF	*β*	P	VIF	*β*	P	VIF	*β*	p
SES	1.112	−0.011	Ns	**	**	**	1.106	−0.068	< 0.01	1.029	−0.075	< 0.001
RELIG	1.071	0.073	< 0.001	1.040	0.068	< 0.01	1.078	0.004	ns	**	**	**
POSITIV	1.677	0.004	Ns	**	**	**	1.677	0.012	ns	**	**	**
KVAL	1.553	−0.023	Ns	**	**	**	1.550	0.057	ns	**	**	**
FRAM	1.277	0.167	< 0.001	1.147	0.176	< 0.001	1.309	−0.063	< 0.05	1.168	−0.063	< 0.01
DEPR	1.611	−0.024	ns	**	**	**	1.605	0.077	< 0.05	1.455	0.124	< 0.001
PRESS	1.588	−0.139	< 0.001	1.311	−0.157	< 0.001	1.588	0.142	< 0.001	1.392	0.146	< 0.001
FYSISK	1.153	0.103	< 0.001	1.106	0.106	< 0.001	1.165	0.035	ns	**	**	**
SPORT	1.067	−0.089	< 0.001	1.047	−0.095	< 0.001	1.078	0.007	ns	**	**	**
COVID	1.230	−0.028	ns	**	**	**	1.226	0.060	< 0.05	1.243	0.059	< 0.05
MOBB	1.505	0.053	ns	**	**	**	1.501	−0.074	< 0.05	1.176	−0.112	< 0.01
SEXTRAK	1.414	−0.105	< 0.001	1.250	−0.094	< 0.001	1.422	0.072	< 0.01	1.388	0.072	< 0.01
FAM1	1.312	0.054	< 0.05	1.191	0.06	< 0.01	1.314	−0.032	ns	**	**	**
FAM2	1.152	0.110	< 0.001	1.127	0.119	< 0.001	1.165	−0.050	< 0.05	1.079	−0.063	< 0.01
VENN	1.381	0.015	ns	**	**	**	1.377	−0.064	< 0.05	1.268	−0.084	< 0.01
BVENN	1.270	−0.061	< 0.01	1.128	−0.075	< 0.001	1.257	0.120	< 0.001	1.225	0.121	< 0.001
SKOLE	1.524	−0.087	< 0.001	1.246	−0.114	< 0.001	1.534	0.005	ns	**	**	**
NAER	1.091	0.055	< 0.05	1.060	0.064	< 0.01	1.094	0.012	ns	**	**	**
NAERT	1.133	−0.069	< 0.01	1.100	−0.063	< 0.01	1.137	−0.037	ns	**	**	**
BALKO	1.653	−0.069	< 0.01	1.396	−0.063	< 0.01	1.644	0.109	< 0.001	1.386	0.098	< 0.001
BROEYK	1.506	0.137	< 0.001	1.249	0.165	< 0.001	1.530	0.008	ns	**	**	**
BDRUG	1.468	0.040	ns	**	**	**	1.468	−0.023	ns	**	**	**
BANTI	1.592	−0.030	ns	**	**	**	1.589	0.066	< 0.05	1.374	0.046	ns
BANTIV	1.439	0.049	ns	**	**	**	1.438	−0.061	ns	**	**	**
BMOBB	1.256	0.005	ns	**	**	**	1.256	−0.011	ns	**	**	**
SMS	1.208	−0.031	ns	**	**	**	–	–	–	–	–	–
DIGI	–	–	–	–	–	–	1.303	−0.033	ns	**	**	**

Notes: **VIF:** The variance inflation factor, used to examine collinearity among the predictor constructs in the structural model. VIF values of 3.3 or higher indicate collinearity issues in this study.

**ns:** Not significant. **: Not included in the final model because *P* > 0.05 in the first model.

Regarding environmental factors, digital media use was positively associated with trust between parents (FAM1, *β* = 0.060), poor relationships with parents (FAM2, *β* = 0.119), and the local area (NAER, *β* = 0.064). Conversely, it was negatively associated with hanging out with friends (BVENN, *β* = −0.075) and perceptions of local safety (NAERT, *β* = −0.063). No associations were observed with peer relationships (VENN).

For risk-taking behaviors, digital media use was positively associated with smoking (BROEYK, *β* = 0.165) and negatively associated with drinking (BALKO, *β* = −0.063). However, no significant associations were found with other risk behaviors, such as drug use (BDRUG) or rule-breaking behavior (BANTI). Additionally, social media use did not exhibit any significant association with digital media use (DIGI).

#### 6.2.2 Social media use

In terms of individual factors, social media use was significantly positively associated with socioeconomic status (SES, *β* = 0.075), depressive thoughts (DEPR, *β* = 0.124), pressure (PRESS, *β* = 0.146), COVID-19 (COVID, *β* = 0.059), and sexual harassment (SEXTRAK, *β* = 0.072). It was negatively associated with future outlook (FRAM, *β* = −0.063) and being bullied (MOBB, *β* = −0.112). No associations were found with religion (RELIG), physical activity (FYSISK), or sport activity (SPORT).

Within environmental factors, social media use was positively associated with hanging out with friends (BVENN, *β* = 0.121) and negatively associated with poor relationships with parents (FAM2, *β* = −0.063) and peer relationships (VENN, *β* = −0.084). No significant relationships were found with factors related to school (SKOLE) or the local area (NAER, NAERT).

For risk-taking behaviors, social media use was significantly positively associated with drinking (BALKO, *β* = 0.098) but not with other risk behaviors, such as smoking (BROEYK) or rule-breaking behavior (BANTI). Furthermore, social media use did not exhibit a significant association with digital media use (DIGI).

### 6.3 Summary of results

Overall, the results indicated that of the 26 paths examined, 14 (53.9 %) were significantly associated with digital media use, and 11 (42.3 %) were significantly associated with social media use. Among these, half of the significant paths related to digital media use (seven out of 14) and over one-third of those related to social media use (four out of 11) demonstrated negative associations. None of the paths related to digital or social media use showed significant associations with positivity (POSITIV), quality of life (KVAL), drug use (BDRUG), aggressive antisocial behavior (BANTIV), or engaging in bullying others (BMOBB).

## 7 Discussion

This study investigated how protective and risk factors are associated with digital and social media use among high school students in Finnmark County, considering individual factors, environmental factors such as family, peers, school, and community, as well as other risk-taking behaviors. The findings of this study revealed nuanced relationships between digital and social media use and various individual, environmental, and behavioral factors.

### 7.1 The distinct patterns of association between digital and social media use

Digital media use was associated with numerous environmental factors, such as relationships with parents, school, and satisfaction with the local area, while social media use was primarily linked to peer-related dynamics. On the other hand, social media use was more strongly related to internal feelings and negative experiences, including being sexually harassed and the impact of COVID-19, whereas digital media use was associated with future outlook and feeling of performance pressures. Additionally, some factors demonstrated divergent association between digital and social media use, including feelings of performance pressure, future outlook, experiences of sexual harassment, negative relationships with parents, and time spent hanging out with friends.

### 7.2 Digital media use: environmental factors

Digital media use was significantly associated with environmental factors, particularly relationships involving parents, showing the significant role of family relationships in shaping adolescents' digital media use, consistent with prior research. Open communication within the family has been shown to reduce the risk of problematic internet use (PIU) [44,45]. Closer parent-child relationships are associated with decreased PIU symptoms over time [20], while overly restrictive parenting practices have limited impact on mitigating PIU [46]. Cultural nuances also influence parenting styles and media use. Aarsand [47] observed that parental views on media reflect broader cultural attitudes toward “good parenting,” which could vary in regions like Finnmark due to its unique sociocultural environment. Despite improvements in parent-child relationships among high school students in Finnmark, these remain significantly lower than the national average [27], suggesting regional factors such as geographic isolation and cultural diversity may shape these dynamics.

Furthermore, satisfaction with the local area was linked to decreased digital media use, suggesting the influence of broader family and community support systems. However, trend analysis in Finnmark revealed that satisfaction with the local area and facilities either worsened or remained unchanged over time for high school students in the region. These students also reported lower satisfaction compared to the national average. This decline may be attributable to the population decrease in many municipalities within Finnmark County [48], leading to the downsizing or closure of youth clubs and culture schools—municipality-owned after-school programs offering music and visual arts classes.

Lower digital media use was associated with increased physical activity, evening socialization with friends, and drinking. This aligns with earlier findings suggesting that face-to-face interactions and physical activity reduce reliance on digital media among high school students in Finnmark [27]. However, the link between reduced digital media use and negative behaviors such as increased drinking warrants further investigation to understand potential compensatory mechanisms. Similarly, since prior research indicated an association between evening socialization with friends and increased drinking behavior [29], this aspect also requires further exploration to identify underlying compensatory mechanisms.

Increased digital media engagement, such as online gaming and video content, is associated with stronger school connectedness. This aligns with prior research indicating that shared digital activities can foster social ties, reduce isolation, and enhance interpersonal communication [23]. Additionally, supportive teacher-student relationships and autonomy-promoting environments are associated with better school engagement and reduced problematic internet use [24]. In Finnmark, while students reported lower overall school connectedness compared to the national average, they expressed higher satisfaction with teachers [27]. This suggests that systemic school challenges, rather than teacher relationships, may contribute to reduced engagement.

Unique to this study is the observed connection between digital media use and feeling of performance pressure and future outlook. This aligns with research suggesting that environmental stressors and aspirations influence adolescents' engagement with technology as a coping mechanism [14].

### 7.3 Social media use: peer dynamics and internal experiences

Social media use was primarily associated with peer dynamics and internal experiences, including exposure to sexual harassment and bullying, and negative peer relationships, though the associations were somewhat mixed. Peer dynamics play a pivotal role in shaping social media behaviors. The findings reveal that social media use is higher among individuals who hang out with friends in the evening, suggesting that peer interaction stimulates online engagement, likely to sustain or extend in-person interactions. This aligns with Liang's study on temporal activity patterns [49], which highlights peer influence as a driver of clustering effects in social media usage. However, the findings also indicate that poor peer relationships are associated with decreased social media use, suggesting that social media functions more as an extension of positive peer interactions rather than a remedy for poor relationships. Similarly, Chen et al. [46] found that lower social support predicts problematic internet use (PIU), emphasizing that social media may be less appealing or beneficial for those with strained peer connections.

Internal experiences such as depression, performance pressure, and negative events like sexual harassment correlate with increased social media use, indicating that social media may serve as a coping mechanism for individuals seeking distraction or validation. Notably, adolescents subjected to cyberbullying often report higher internet use [19], pointing to a paradoxical dynamic where social media becomes both a refuge and a risk factor. Conversely, a negative future outlook and experiences of being bullied are associated with reduced social media use, indicating that the emotional burden of bullying or a pessimistic perspective on the future may inhibit engagement in online interactions.

The dual role of social media as both a facilitator and detractor of socialization is evident. Willoughby's findings [20] suggest that higher friendship quality predicts greater internet use, whereas low peer support fosters PIU [46]. Thorsteinsson and Davey's research [50] underscores this duality, showing that social use of the internet can mitigate future problematic usage, contrasting with non-social online activities. In regions like Finnmark, where students report having fewer trusted friends [27], the protective role of social media in fostering peer connections might be undermined by broader societal and cultural factors. Sámi youth facing discrimination [51,52] may encounter compounded challenges in harnessing the potential benefits of social media.

Regional trends in Finnmark provide a poignant context for understanding these dynamics. Increased social media use, paired with declining physical activity and higher depressive thoughts and exposed to bullying [27] underscores the need for targeted interventions, particularly to address cyberbullying and discrimination.

### 7.4 Risk behavior

The relationship between risk behaviors and digital/social media use is nuanced, with limited associations observed. Smoking correlates with increased digital media use, and drinking is associated with higher social media engagement, potentially reflecting the social context of these behaviors. However, antisocial behaviors and bullying others show no significant links to media use.

Alcohol-related content on social media may reinforce drinking behaviors. Heavy drinkers are influenced by their networks, which often include drinking peers, while light drinkers encounter glamorized depictions of alcohol use [53]. Smoking's association with digital media use is less explored. In Finnmark, smoking and drinking behaviors have declined in line with international trends [54], while antisocial behaviors have risen. Bullying remains a persistent issue [55,56], particularly cyberbullying. These findings highlight the complex interplay of individual, social, and regional factors in shaping risk behaviors and media use.

### 7.5 Contrasts and non-associations

The lack of direct associations between digital and social media use, as well as their differing relationships with specific factors, highlights the distinct nature of these platforms. Digital media use appears more tied to environmental influences, while social media use aligns with interpersonal and emotional dimensions.

Given the distinct patterns of associations between digital and social media use, prevention strategies should be tailored separately for these forms of media. Social media use interventions should focus on mitigating its links to negative internal experiences and peer dynamics, while strategies for digital media use should address its connections to environmental and behavioral factors.

### 7.6 Lack of positive internal associations

Neither digital nor social media use showed significant associations with positive internal feelings, such as quality of life or overall positivity. This finding contrasts with the participatory and moral online behavior fostered by parental mediation and peer pressure [12]. The absence of positive associations in this study may suggest that other mediating factors—such as digital literacy or context-specific parental involvement—are necessary to realize such benefits.

Additionally, the absence of significant relationships between social media use and positive internal feelings, such as quality of life, contrasts with earlier studies suggesting potential benefits of online connections [4,5]. This discrepancy may reflect the regional and sociocultural context of Finnmark, emphasizing the importance of localized studies in understanding these dynamics.

### 7.7 Clinical and intervention implications

The study highlights the importance of addressing environmental stressors (e.g., performance pressure) and emotional challenges (e.g., depression, harassment) when addressing adolescents' media use. Social media's dual role as both a coping mechanism and a risk factor requires careful assessment and intervention. In regions like Finnmark, where bullying and depressive symptoms are prevalent, clinicians should account for broader societal and cultural influences.

From a public health perspective, digital and social media use must be understood as key determinants of adolescent well-being. Digital media use is strongly influenced by family dynamics and school connectedness, whereas social media use is more closely tied to peer interactions and mental health risks. These findings align with broader research suggesting that while digital platforms facilitate social connectivity and access to information, they also contribute to mental health challenges, cyberbullying, and misinformation exposure. To mitigate risks and enhance protective factors, public health interventions should prioritize the following: 1) digital media interventions to strengthen family and community support, promoting physical activity, and improving local area satisfaction; and 2) social media interventions to address peer dynamics, bullying, and emotional well-being, while fostering digital literacy. In Finnmark, localized efforts targeting cyberbullying, discrimination, and mental health support are critical to mitigating risks associated with media use.

### 7.8 Limitation

The present study had several limitations that should considered. First, the Ungdata survey is school based, which excludes students who have dropped out of school. Second, the response rate of high school students was lower than that of junior high students, with only around 66–68 % of high school students responding, compared to 80–83 % of junior high students. This lower response rate may have introduced some bias in the results.

Additionally, the survey used cross-sectional data, preventing the establishment of causal inferences or temporal relationships between the studied variables. Some questions were answered retrospectively, making them susceptible to recall bias and memory lapses.

Another limitation is the inconsistency in the time frames used to assess variables. Media use had no specific time frame, antisocial behavior and drug use were assessed over the past year, and depression was measured over the past week. The timing of data collection, such as during exams or emotionally stressful periods, may have influenced results, especially for variables like depression, which are assessed over shorter time frames.

The variables related to digital media use (such as online gaming and YouTube watching) and social media use in this study primarily focused on the frequency and duration of media use, without incorporating variables describing user behavior. Consequently, it was not possible to determine whether the media use was problematic, how much time was spent on problematic media use, or whether it was related to cyberbullying. This limitation may explain the high percentage of negative associations observed in both digital and social media use: half of the significant paths for digital media use (seven out of 14) and four out of 11 paths for social media use. Future research should include behavioral variables to better understand and address the risks associated with media use among adolescents.

A statistical limitation involves the treatment of outliers in the PLS-SEM analysis. While PLS-SEM is robust to non-normal data and does not require strict distributional assumptions, many studies do not explicitly test for outliers [57]. However, influential outliers can still bias parameter estimates and affect model accuracy [39]. Additionally, the lack of direct associations between DIGI and SMS suggests potential moderation, mediation, or nonlinear interactions that were not explored. Although steps were taken to ensure no multicollinearity, these interaction effects were not explicitly tested in the current model.

Lastly, the survey did not include indicators related to ethnicity. Given the distinct socio-cultural and multi-ethnic contexts of Finnmark County, particularly the presence of the Sámi and Kven people, as well as individuals from Northern Finland, Northern Sweden, and Russia, and the inter-marriage of these groups, the diversity within these populations may have influenced factors affecting adolescents, such as family, social networks, health, and risk behaviors. Different cultures and different group of people shape individuals' behavioral decisions in various ways. Social norms—such as perceptions of what others do and expect—vary across cultural and social networks, affecting perceived health and well-being and risk behaviors [58,59]. Future studies should include ethnicity-related factors, such as ethnic identity, to better understand these issues.

## 8 Conclusion

This study explored the relationship between protective and risk factors in digital and social media use among high school students in Finnmark County. The study highlights the need to distinguish between digital and social media use, given their unique associations with individual, environmental, and behavioral factors. Digital media use was primarily associated with environmental influences, including family relationships, school connectedness, and satisfaction with the local area. In contrast, social media use was more strongly related to peer dynamics, internal feelings, and negative experiences, including depression and sexual harassment.

From a public health perspective, these findings underscore the broader implications of adolescent media use for mental health, social well-being, and risk behaviors. Regional factors in Finnmark, such as geographic isolation and cultural diversity, shape adolescents' media use behaviors. For instance, lower satisfaction with local areas and facilities, combined with the decline in physical activity, suggests that adolescents in Finnmark might be increasingly turning to digital media as a form of social connection and emotional coping. Furthermore, the prevalence of bullying and the rising mental health challenges underscore the need for interventions targeting the negative aspects of social media use emphasize the public health necessity of targeted interventions that mitigate the negative effects of social media while fostering its potential benefits.

Future research should further explore the behavioral aspects of media use, such as the intensity and quality of engagement, to better understand the potential risks associated with problematic internet use. It is also crucial for future studies to incorporate ethnicity-related factors, particularly given the diverse cultural makeup of Finnmark, including the Sámi, Kven, and other indigenous groups. Addressing these dimensions will contribute to a more comprehensive public health approach to adolescent digital and social media use, ensuring that interventions are culturally responsive and regionally relevant.

## CRediT authorship contribution statement

**Shiho Hansen:** Writing – review & editing, Writing – original draft, Validation, Project administration, Methodology, Investigation, Funding acquisition, Formal analysis, Data curation, Conceptualization.

## Declaration of generative AI and AI-assisted Technologies in the Writing Process

During the preparation of this work the author used ChatGPT-4o mini, in order to improve the readability and language of the manuscript, as well as to check grammar.

## Funding

This research was partially supported by the internal research fund of 10.13039/501100012251Finnmark Hospital Trust. None of the funding providers had any role in the data analyses and interpretation, nor had they any right to approve or disapprove the writing and publication of the manuscript.

## Declaration of competing interest

The author declares that they have no known competing financial interests or personal relationships that could have appeared to influence the work reported in this paper.

## Data Availability

The data for this study are available from the Norwegian Agency for Shared Services in Education and Research (Sikt). Restrictions apply to the availability of these data, which were used under license for this study. Access to the dataset can be requested via https://surveybanken.sikt.no/no/study/7ae7f902-ddde-4bd8-8311-21f16ce93fdf/undefined?type=studyMetadata&datafile=9c72d168-67ae-4c15-82d9-738535771af9/4&elements=[. The dataset may be used for research purposes with the approval of Sikt and Norwegian Social Research (NOVA).

## References

[1] American Psychological Association (2023).

[2] Orben A., Tomova L., Blakemore S.J. (2020). The effects of social deprivation on adolescent development and mental health. Lancet Child Adolesc Health.

[3] Kanchan S., Gaidhane A. (2023). Social media role and its impact on public health: a narrative review. Cureus.

[4] Reid Chassiakos Y. (2016). Children and adolescents and digital media. Pediatrics.

[5] Reich S.M., Subrahmanyam K., Espinoza G. (2012). Friending, IMing, and hanging out face-to-face: overlap in adolescents’ online and offline social networks. Dev Psychol.

[6] Borca G. (2015). Internet use and developmental tasks: adolescents’ point of view. Comput Hum Behav.

[7] Kranzler E.C., Bleakley A. (2019). Youth social media use and health outcomes: #diggingdeeper. J Adolesc Health.

[8] Anderson C.A. (2010). Violent video game effects on aggression, empathy, and prosocial behavior in eastern and western countries: a meta-analytic review. Psychol Bull.

[9] Keles B., McCrae N., Grealish A. (2020). A systematic review: the influence of social media on depression, anxiety and psychological distress in adolescents. Int J Adolesc Youth.

[10] UNICEF (2024). Cyberbullying: What is it and how to stop it. https://www.unicef.org/end-violence/how-to-stop-cyberbullying.

[11] WHO/Europe (2024).

[12] Coyne S.M. (2017). Parenting and digital media. Pediatrics.

[13] Festl R. (2021). Social media literacy & adolescent social online behavior in Germany. J Child Media.

[14] Anderson E.L., Steen E., Stavropoulos V. (2017). Internet use and problematic internet use: a systematic review of longitudinal research trends in adolescence and emergent adulthood. Int J Adolesc Youth.

[15] Symons K. (2020). Parents’ concerns over internet use, their engagement in interaction restrictions, and adolescents’ behavior on social networking sites. Youth Soc.

[16] Sampasa-Kanyinga H. (2020). Social media use and parent-child relationship: a cross-sectional study of adolescents. J Community Psychol.

[17] Kanter M., Afifi T., Robbins S. (2012). The impact of parents “friending” their young adult child on facebook on perceptions of parental privacy invasions and parent–child relationship quality. J Commun.

[18] Gunuc S. (2017). Peer influence in internet and digital game addicted adolescents: is internet / digital game addiction contagious?. Int J High Risk Behav Addict.

[19] Gámez-Guadix M. (2014). Depressive symptoms and problematic internet use among adolescents: analysis of the longitudinal relationships from the cognitive-behavioral model. Cyberpsychol Behav Soc Netw.

[20] Willoughby T. (2008). A short-term longitudinal study of internet and computer game use by adolescent boys and girls: prevalence, frequency of use, and psychosocial predictors. Dev Psychol.

[21] Leijse M.M.L., Koning I.M., van den Eijnden R.J.J.M. (2023). The influence of parents and peers on adolescents’ problematic social media use revealed. Comput Hum Behav.

[22] Soh P.C.-H. (2018). Parents vs peers’ influence on teenagers’ internet addiction and risky online activities. Telematics Inform.

[23] Stavropoulos V. (2017). MMORPG gaming and hostility predict internet addiction symptoms in adolescents: an empirical multilevel longitudinal study. Addict Behav.

[24] Yu C., Li X., Zhang W. (2015). Predicting adolescent problematic online game use from teacher autonomy support, basic psychological needs satisfaction, and school engagement: a 2-year longitudinal study. Cyberpsychol Behav Soc Netw.

[25] World Health Organization (2022).

[26] Statistisk sentralbyrå (2023).

[27] Hansen S. (2024). The trends in perceived health, well-being, and risk behaviours among high school students in Finnmark, Norway, compared to the national average. Int J Circumpolar Health.

[28] Frøyland L.R., Bakken A., von Soest T. (2020). Physical fighting and leisure activities among Norwegian adolescents-investigating co-occurring changes from 2015 to 2018. J Youth Adolesc.

[29] Hansen S. (2025).

[30] Rushfeldt R. (2001).

[31] Trepte S., Masur P.K. (2016).

[32] Alsaleh D.A. (2019). Cross-cultural differences in the adoption of social media. J Res Interact Mark.

[33] Jackson L.A., Wang J.-L. (2013). Cultural differences in social networking site use: a comparative study of China and the United States. Comput Hum Behav.

[34] Bronfenbrenner U. (1979).

[35] Ungdatasenteret (2024). Ungdata. https://www.ungdata.no/.

[36] Ungdatasenteret (2020).

[37] Ungdatasenteret and KoRus-Nord (2021).

[38] Sikt (2010).

[39] Hair J.F. (2021).

[40] Kock N., Hadaya P. (2018). Minimum sample size estimation in PLS-SEM: the inverse square root and gamma-exponential methods. Inf Syst J.

[41] Ringle C.M. (2023). A perspective on using partial least squares structural equation modelling in data articles. Data Brief.

[42] Henseler J., Ringle C.M., Sarstedt M. (2015). A new criterion for assessing discriminant validity in variance-based structural equation modeling. J Acad Mark Sci.

[43] Diamantopoulos A., Siguaw J.A. (2006). Formative versus reflective indicators in organizational measure development: a comparison and empirical illustration. Br J Manag.

[44] van den Eijnden R.J. (2010). Compulsive internet use among adolescents: bidirectional parent-child relationships. J Abnorm Child Psychol.

[45] Yu L., Shek D.T. (2013). Internet addiction in Hong Kong adolescents: a three-year longitudinal study. J Pediatr Adolesc Gynecol.

[46] Chen Y.L., Chen S.H., Gau S.S. (2015). ADHD and autistic traits, family function, parenting style, and social adjustment for internet addiction among children and adolescents in Taiwan: a longitudinal study. Res Dev Disabil.

[47] Aarsand P. (2011). Parenting and digital games. J Child Media.

[48] Statistisk sentralbyrå (2024).

[49] Liang H., Shen F. (2018). Birds of a schedule flock together: social networks, peer influence, and digital activity cycles. Comput Hum Behav.

[50] Thorsteinsson E.B., Davey L. (2014). Adolescents’ compulsive internet use and depression: a longitudinal study. Open J Depres.

[51] Bals M. (2010). Internalization symptoms, perceived discrimination, and ethnic identity in indigenous Sami and non-Sami youth in Arctic Norway. Ethn Health.

[52] Lenert K., Sara H., Skaar W. (2021).

[53] Curtis B.L. (2018). Meta-analysis of the Association of Alcohol-Related Social Media use with alcohol consumption and alcohol-related problems in adolescents and young adults. Alcohol Clin Exp Res.

[54] Raitasalo K. (2021). Similar countries, similar factors? Studying the decline of heavy episodic drinking in adolescents in Finland, Norway and Sweden. Addiction.

[55] Henriksen A.B. Child in northern Norway are bullied the most [barn i Nord-Norge mobbes mest]. Klikk 2017. [cited 2024 Dec 28]. https://www.klikk.no/foreldre/barn-i-nord-norge-mobbes-mest-2352498.

[56] NRK (2023). NRK Troms og Finnmark.

[57] Sarstedt M. (2022). Progress in partial least squares structural equation modeling use in marketing research in the last decade. Psychol Mark.

[58] Bicchieri C. (2017).

[59] Cialdini R.B., Trost M.R., Gilbert D.T., Fiske S.T., Lindzey G. (1998). The handbook of social psychology.

